# Experimental analysis of genetic and environmental interactions on leaf elongation and reproductive development in *Lolium perenne*

**DOI:** 10.1093/aobpla/plae069

**Published:** 2024-12-24

**Authors:** Simon Rouet, Jean-Louis Durand, Alice Troux, Romain Barillot

**Affiliations:** INRAE, URP3F, 86600 Lusignan, France; INRAE, URP3F, 86600 Lusignan, France; INRAE, URP3F, 86600 Lusignan, France; INRAE, URP3F, 86600 Lusignan, France

**Keywords:** *Lolium perenne*, photoperiod, temperature, floral induction, heading, grasslands, leaf growth, phenology

## Abstract

Perennial grasses’ reproductive phenology profoundly impacts plant morphogenesis, biomass production, and perenniality in natural ecosystems and cultivated grasslands. Complex interactions between vegetative and reproductive development complicate grass phenology prediction for various environments and genotypes. This work aims to analyse genetic × environment interactions effects on tiller growth and reproductive development in *Lolium perenne.* Three perennial ryegrass cultivars, Bronsyn, Carvalis, and Tryskal, were grown from seedling to heading under four inductive conditions. T0 plants were continuously exposed to high temperatures and long days (HT–LD). T1, T2, and T3, plants were initially exposed to low temperatures and short days (LT–SD) for 9 weeks. Then, T1 plants were immediately transferred to high temperatures and long days (HT–LD). Before their exposure to HT–LD, T2, and T3 plants were first transferred to high temperatures and short days (HT–SD) for 3 and 6 weeks, respectively. Leaf length, leaf emergence, and heading were regularly monitored. Floral transition and heading only occurred in T1, T2, and T3, i.e. after successive exposure to low temperature and long photoperiod. Bronsyn had higher heading earliness and proportion of reproductive tillers than Carvalis and Tryskal. The duration of HT–SD exposure affected the final number of leaves and spikelets. The rate of leaf and spikelet production significantly increased once plants were exposed to LD. Our results suggest an additive effect of the photoperiod and floral transition on leaf elongation rate. These findings enhance our understanding of the genetic × environment interactions on the vegetative and reproductive development in perennial ryegrass.

## Introduction

The reproductive phenology of perennial grasses encompasses a sequence of seasonal events, starting with the floral transition of the tiller apex, progressing to spike heading, flowering, and culminating in seed dispersal (Heide 1994). This phenology determines plant fitness in natural grasslands and profoundly impacts biomass production rate, its digestibility by ruminants, the harvest season, and the perenniality of cultivated grasslands (Rouet *et al.* 2021). Recent studies by Blanco-Pastor *et al.* (2019, 2021) and Keep *et al.* (Keep *et al.* 2020, 2021) revealed significant associations between the geographical distribution of perennial ryegrass populations in Europe and molecular markers pertained to phenology, including SNPs located in vernalization genes. However, there’s a dearth of quantitative data on the response of reproductive phenology to environmental factors and the genetic variability of these responses, typically high in perennial grasses. This knowledge gap complicates the construction of models for predicting key phenological events in diverse environments and across a broad genotype panel (Rouet *et al.* 2021, 2022).

Perennial grasses are monocarpic, with the irreversible floral transition occurring at the level of each individual tiller over multiple growing seasons (Gillet 1980; Heide 1994). In *Lolium perenne*, most studies indicate that the floral transition necessitates two successive induction phases, each controlled by distinct environmental factors (Heide 1994; Rouet *et al.* 2021). Primary induction is achieved after several weeks of exposure to low temperatures and short photoperiods, while secondary induction is attained after a few days of exposure to high temperatures and long photoperiods. Various combinations of temperature, photoperiod, and exposure duration have been examined to identify the environmental conditions conducive to floral induction (Heide 1994; Aamlid *et al.* 2000). These experiments are instrumental in demonstrating the existence of photoperiodic and thermal thresholds. More recently, important advances were made on the genetic (e.g. *VRN* gene family, *MADS-box*, *PPD1*, and *FT1*) and hormonal (e.g. auxin, cytokinin, and gibberellins) control of floral induction (Colasanti and Coneva 2009; Chen *et al.* 2010; Pautler *et al.* 2013; Zhang and Yuan 2014; Nuñez and Yamada 2017).

Spike heading, which occurs in late spring, is the initial stage of reproductive phenology that can be readily observed with the naked eye. As such, the heading date is a frequently measured variable, particularly used to rank the earliness of species, cultivars, or genotypes. In grasses, the heading date is a result of the combined duration of floral induction and the growth of reproductive organs, leading to the spike’s emergence from the pseudostem (Gillet 1980; Thomas and Evans 1990). Before the floral transition, the tiller is vegetative and the apex produces vegetative organs, including a leaf, an internode, and a lateral apex. During these vegetative stages, the rate of leaf emergence and leaf dimensions determine the pseudostem length from which the spike will emerge. After the floral transition, the apex produces reproductive phytomers, consisting of a spikelet and an internode, derived from a structure common to leaves. Therefore, the heading date depends not only on the activity and stage of the apex but also on the growth processes occurring during both the vegetative (pseudostem length) and reproductive (number of spikelets and peduncle length) stages. As a result, the duration of the floral transition emerges as a key factor influencing both vegetative and reproductive morphogenesis.

During the floral transition, perennial grasses like *L. perenne* typically experience a significant increase in biomass production due to accelerated leaf elongation (Ryle 1966; Davies 1971; Parsons and Robson 1980; Barre *et al.* 2015). Parsons and Robson (1980) demonstrated that this acceleration correlates with a change in the leaf elongation’s intrinsic response to temperature, aligning with the initial stages of floral transition. The causal link between floral induction and increased leaf elongation has been a topic of debate for years. Some researchers hypothesise that the photoperiod increase in early spring directly influences leaf elongation (Ryle 1966; Davies 1971; Hay 1990; Wu *et al.* 2004). In this scenario, leaf elongation acceleration would be a direct response to the photoperiod, which also triggers the floral transition of primary-induced apices concurrently.

The objective of this study is to analyse the effects of genetic and environmental interactions on leaf elongation and reproductive development in *L. perenne*. For this purpose, we monitored leaf production, leaf elongation, and heading dates in three cultivars of perennial ryegrass under varying temperature and photoperiod regimes. Specifically, we examined (i) the intricate interactions between photoperiod, floral transition and leaf elongation rate, (ii) the relationship between the rates of leaf emergence and primordia production during the reproductive phase and (iii) the interaction between genetic variability and environmental conditions on reproductive phenology.

## Materials and methods

The fundamental premise of the experiment was to cultivate three perennial ryegrass cultivars, Bronsyn, Carvalis, and Tryskal, from seedling to heading, under varying durations of exposure to inductive conditions (Supplementary Fig. S1). Four inductive treatments were established, with temperature, photoperiod, and phase duration as factors. These treatments varied in the duration of a non-inductive period (i.e. elevated temperature and short days) applied between the conclusion of primary induction and the commencement of secondary induction. Consequently, plants were anticipated to remain in a vegetative stage for diverse durations before the onset of secondary induction. Throughout the main tiller development, leaf length, emergence rate, and heading date were consistently monitored.

### Plant material

The experiment involved three *L. perenne* cultivars, Bronsyn, Carvalis, and Tryskal, each differing in heading earliness. These cultivars, registered in the French catalogue, were characterized by the French variety and seed study and control group (GEVES). The main information about these cultivars is provided in Table 1. Bronsyn is an early cultivar, Carvalis exhibits intermediary earliness, while Tryskal is a late cultivar. All cultivars are suitable for cultivation in temperate areas and are considered to require winter conditions (cold and short days) to achieve floral transition. Indeed, in the GEVES national test network, no heading was reported in the year following early spring sowing, whereas heading invariably occurred after at least one winter (Rouet *et al.* 2021).

**Table 1.** Details of the cultivars of *Lolium perenne* used in the experiment.

Cultivar	Country	Registration year	Ploidy	Main use	Onset of growth season	Heading date	Flexibility (days)	Aftermath heading
Bronsyn	New-Zealand	2001	2n	Forage	28th March	5th May	38.7	4.2
Carvalis	France	2010	2n	Forage	4th April	22nd May	55.1	2.4
Tryskal	France	2010	2n	Forage	10th April	6th June	65.5	2.1

Spring growth, heading date, flexibility (number of available days for grassland exploitation), and aftermath heading (score between 1 and 9, with 9 corresponding to a cultivar with a significant aftermath heading) represent the average of observations made across different sites and years. *Source*: Herbe Book, https://www.herbe-book.org/ 2019 edition.

The experiment aimed to study the effects of environmental conditions on the reproductive phenotype and thus began with growing plants from seeds. This ensured uniform exposure to environmental conditions and age across all tillers. The seeds, provided by seed companies and harvested in the summer of 2018, were stored in dark, room-temperature conditions to prevent primary induction.

### Growth conditions

Initially, seeds were germinated in Petri dishes in the dark at an average temperature of 18°C. After four days, seedlings were sown in peat microsods and placed in a greenhouse for four weeks in November 2018. The air temperature was maintained above 15°C and light was added in the morning and evening to ensure a 13-h photoperiod (using 400 W sodium lamps) to prevent primary induction.

Following this initial growth phase, the plants were transplanted into 3L plastic pots filled with a substrate composed of 60% PELTRACOM loam, 30% superficial soil from Lusignan, France, and 10% sand and a controlled-release fertilizer (12/14M; N-P-K: 14-18-11). The plants were arranged in a randomized block design in two growth chambers (model 97132/7NU, Froids et Mesures, Beaucouzé, France), each with a specific temperature and day length setup. Depending on the induction treatment, the plants were progressively transferred from one chamber to another. When all plants were gathered in the same growth chamber, the density was 70 plants m^−2^. Both chambers were irrigated to ensure optimal growth conditions. The light was provided by HQI lamps (POWERSTAR, HQI-T 400WD lamps, OSRAM, Munich, Germany) with a photosynthetic photon flux density of 240 µmol m^−2^ s^−1^ at the top of the canopy.

### Temperature and photoperiodic treatments

In the experiment, plants were exposed to four distinct inductive conditions in growth chambers, each with varying durations and levels of temperature and photoperiod. Chamber 1 maintained ‘high temperature and long day’ conditions (HT–LD: 18°C–16 hours) throughout the experiment. In contrast, Chamber 2 initially set ‘low temperature and short day’ conditions (LT–SD: 6°C–8 hours), transitioning later to ‘high temperature and short day’ (HT–SD: 18°C–8 hours).

**Figure 1.**
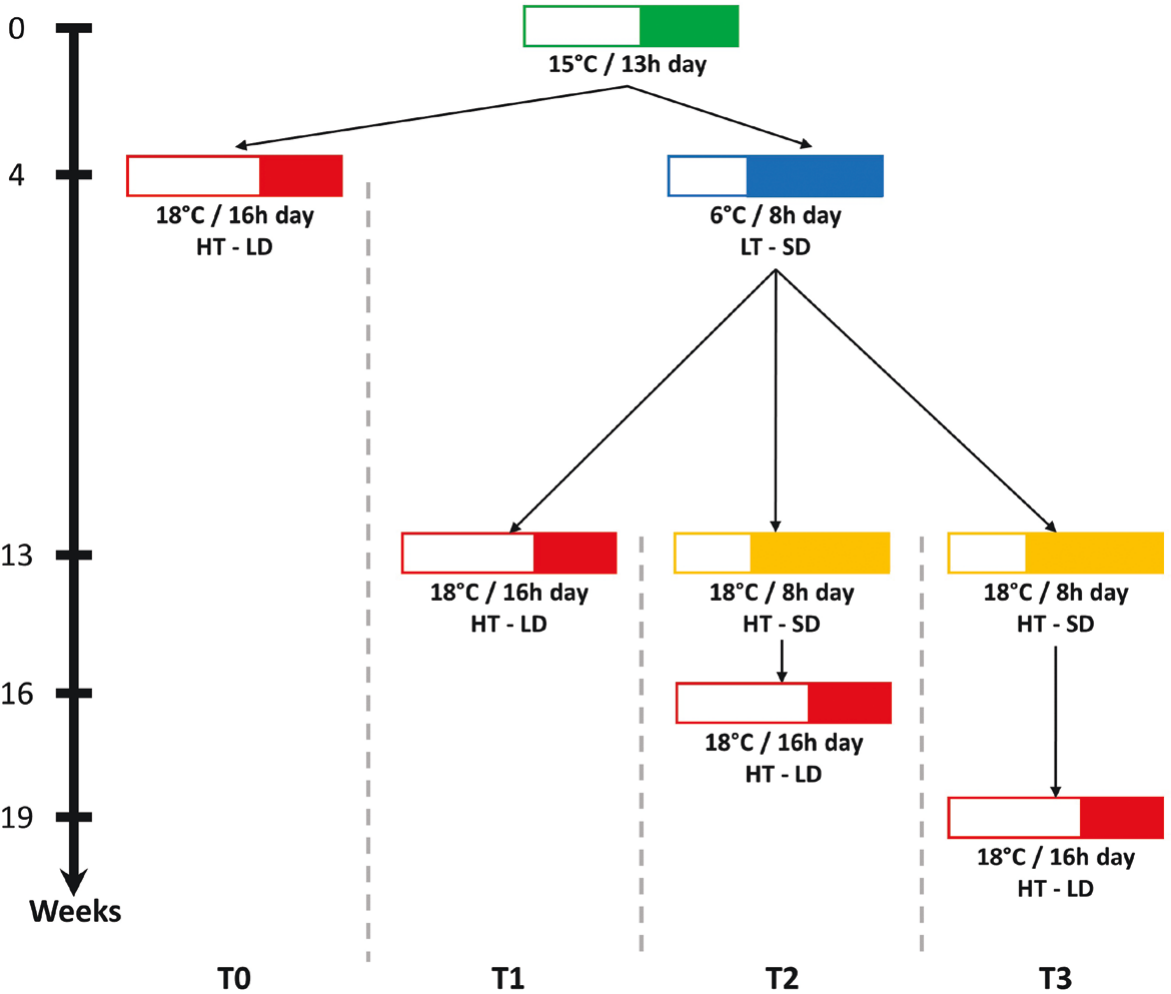
Diagram illustrating the inductive treatments applied to *Lolium perenne* cultivars. Plants were initially grown in a greenhouse (green rectangle) and subsequently transferred into two growth chambers. Plants of T0 remained in high temperatures (HT, 18°C) and long days (LD, 16 hours) throughout the experiment (red rectangle). Plants of T1, T2, and T3 were first exposed to low temperatures (LT, 6°C) and short days (SD, 8 hours) for 9 weeks (blue rectangle). Plants of T1 were immediately transferred to HT–LD conditions. Plants of T2 and T3 were exposed to HT and SD (yellow rectangles) for 3 and 6 weeks, respectively, before being transferred to HT and LD conditions.

Treatment 0 (T0) involved 78 plants (26 per cultivar) continuously exposed to the HT–LD conditions of chamber 1 (Fig. 1). This treatment served as a control, as the HT–LD conditions were expected to prevent the flowering transition due to the absence of cold temperature exposure. The other treatments (T1, T2, and T3), initially exposed all plants (26 per cultivar) to conditions designed to trigger floral induction. Thus, plants in these treatments were first exposed to LT–SD conditions for 9 weeks in Chamber 2, expected to fulfil the primary induction requirement (Aamlid *et al.* 2000; Paina *et al.* 2014). Subsequently, the temperature in Chamber 2 was raised to 18°C, while maintaining a short photoperiod (HT–SD). Plants in T1, T2, and T3 were successively transferred from Chamber 2 (HT–SD) to Chamber 1 (HT–LD) with varying delays. T1 plants were transferred to HT–LD immediately after the 9-week exposure to LT–SD, implying no delay between the completion of primary induction and exposure to long days. In contrast, T2 and T3, plants remained under HT–SD conditions in Chamber 2, for 3 and 6 weeks, respectively, before being transferred to HT–LD (Chamber 1). The long day conditions mirrored those used by Paina *et al.* (2014), except for a slightly lower temperature (18°C instead of 20°C).

In Chamber 1, an 8 hours photoperiod extension was implemented using LED tubes (Philips, MAS LED tube UN 1500 mm UO 24W840 T8) with 75% of the surface covered. These tubes were activated for 4 hours before and after the HQI lamps were switched on and off, respectively. The additional irradiance from the LED tubes was low enough (+8 µmol m^−2^ s^−1^ of PPFD) to ensure that the increase in energy supply was not significant compared to plants under short day conditions.

### Measurements

Starting from the transplanting date (4 weeks post-sowing), the visible length of each leaf (from the tip of the lamina to the top of the previous leaf sheath) was measured on the main tiller of each plant every 2 or 3 days with a 1 mm precision ruler. To evaluate the inductive treatments effects on leaf elongation, the results are expressed as ‘tiller elongation’, defined as the daily cumulative elongation of all visible leaves on a tiller. Leaf elongation measurements ceased when internode elongation began, as it was impossible to differentiate between internode and sheath elongation non-destructively. Additionally, the date of ligule emergence out of the previous leaf sheath was recorded for each leaf in order to calculate the rate of leaf ligulation. Lastly, the heading date, identified as the date when the first spikelet emerged from the pseudostem, was monitored.

Once the tillers had ceased growing for several days and the spikes had fully emerged, the tillers were gathered to count the number of spikelets. However, some plants prematurely perished between the time of spike heading and full maturity, precluding the measurement of spikelets for every headed plant. The length of long internodes (i.e. exceeding 1 mm) and peduncles were measured exclusively for T1 plants (Supplementary Fig. S2). At the experiment’s conclusion, the main tillers without an emerged spike were dissected to inspect the apex and verify the absence of floral transition. To that purpose, shoot apices were isolated from the surrounding leaves under a stereoscopic binocular microscope in order to determine their phenological stage. We looked for typical morphological markers of floral transition in grasses such as elongated apices, double ridges, or white stripes (Gonthier and Francis 1989; Heide 1994; Rouet *et al.* 2021).

### Statistical analyses

The analysis of variance and nonlinear regression were conducted using R software (R Core Team 2021). The analysis of variance (ANOVAs) followed a two-factor linear model, represented as


$$\begin{array}{l} y_{ij}=\mu +\;T_{i}+V_{j}+\;\gamma_{ij}+\varepsilon_{ij} \end{array}$$

Here, *y*_*ij*_ is any measured variable for treatment (*i, j*), *μ* represents the mean value of *y*, *T* signifies the additive main effect of the inductive treatment *i, V* signifies the main additive effect of the variety *j*, *γ* is the nonadditive interaction effect of treatment (*i, j*), and *ε* is the random error.

The normal distribution of residuals from ANOVAs was verified using the Shapiro–Wilk test. Homoscedasticity was assessed by examining the random distribution of the residuals. For values not normally distributed, median comparisons were performed using the Wilcoxon–Mann–Whitney test. In all instances, the H_0_ was rejected when the *P*-value was less than 0.05. In the results section, the displayed values are the median values along with the associated standard error. Boxplots were generated using the Tukey style, which includes the range from (−1.5 * InterQuartile Range, the 2nd quartile, the median, and the 3rd quartile, +1.5 * IQR).

## Results

### Heading date

**Figure 2.**
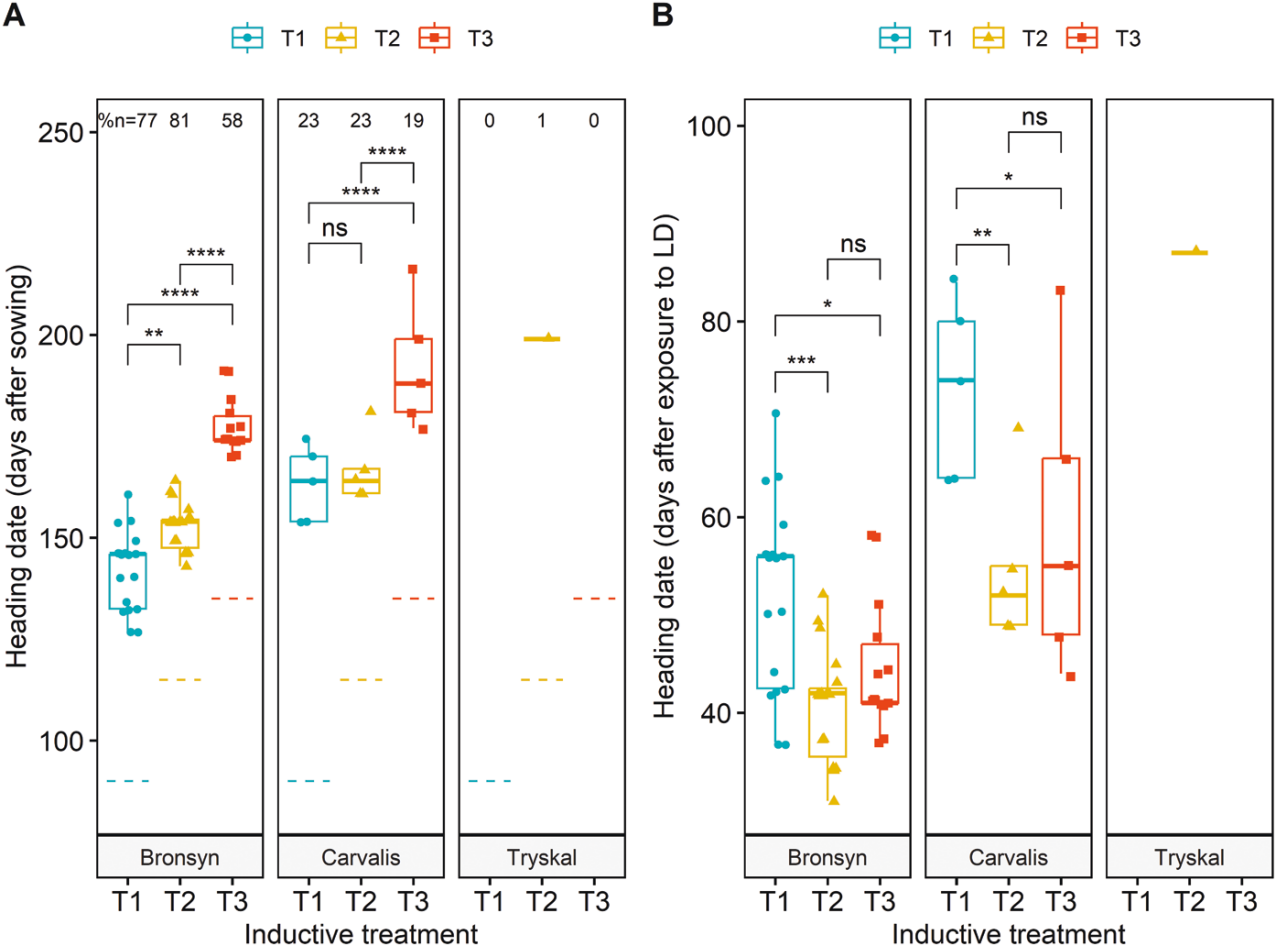
Heading dates in cultivars Bronsyn, Carvalis, and Tryskal were exposed to the different inductive treatments T1 (blue circles), T2 (yellow triangles), and T3 (red squares).(A) Heading date calculated from sowing. (B) Heading date calculated from the exposure to long photoperiods. The percentage of headed plants for each treatment is indicated above each boxplot in A. The start of exposure to long days is indicated by dashed lines in A for each treatment. Statistical differences between treatments are shown in A (ns: non-significant, *: *P* < 0.05, **P < 0.01, ***P < 0.001, ****P < 0.0001).

As anticipated, none of the plants from T0 reached the heading stage during the experiment i.e. after 180 days post-sowing and 80 days under inductive conditions (Table 2). This outcome affirmed that the tested cultivars required winter-like conditions to achieve floral transition. For Bronsyn, 77%, 81%, and 58% of the plants underwent floral transition and produced a spike in T1, T2, and T3, respectively (Fig. 2A). Conversely, Carvalis, had a significantly lower percentage of headed plants (23% in T1, 23% in T2, and 19% in T3). Interestingly, only one plant of the Tryskal cultivar underwent the floral transition and reached the heading stage. This was corroborated by the tiller dissection of each non-headed plant, which revealed no signs of floral transition at the apex level. As a result, the Tryskal cultivar was excluded from the subsequent analyses of reproductive development, but various hypotheses to explain this behaviour are proposed in the discussion. A comparison of heading dates between Bronsyn and Carvalis cultivars from the same treatment revealed that Bronsyn consistently headed earlier than Carvalis (*treatment T1: W = 15, P < 0.01, treatment T2: W = 13.5, P < 0.01, treatment T3: W = 14, P < 0.05*) (Fig. 2A). This ranking aligned with the observations made by the GEVES group under natural conditions (Table 1). The delay between the heading dates of the two cultivars was 21 days in T1, 12 days in T2, and 14 days in T3. There was no significant interactive effect between the varieties and the treatments (*P = 0.46*).

**Table 2.** Effect of inductive treatments on heading, leaf, and spikelet production in Bronsyn, Carvalis, and Tryskal (mean ± SD).

	Bronsyn				Carvalis				Tryskal			
	T0	T1	T2	T3	T0	T1	T2	T3	T0	T1	T2	T3
Headed plants (%)	0	77	81	58	0	23	23	19	0	0	1	0
Heading date (days after sowing)	–	142 ± 10	153 ± 6	178 ± 7	–	163 ± 9	167 ± 8	192 ± 16	–	–	199	–
Heading date (days after LD)	–	52 ± 10	41 ± 6	45 ± 7	–	73 ± 9	55 ± 8	59 ± 16	–	–	87	–
Final leaf number*	16.5 ± 2.5	14.05 ± 1.6	14.5 ± 1.0	17.3 ± 1.4	14.7 ± 2.1	15.6 ± 0.9	16.3 ± 1.5	17.5 ± 0.7	15.2 ± 2.9	–	20	–
Emerged leaves after LD exposure	–	8.5 ± 1.4	5.7 ± 0.7	5.9 ± 1.4	–	9.8 ± 0.8	7.3 ± 1.5	7.0 ± 0	–	–	8	–
Emerged leaves at LD exposure	–	5.5 ± 0.5	8.7 ± 0.6	11.3 ± 0.8	–	5.8 ± 0.4	9 ± 0	10.5 ± 0.7	–	–	12	-
Number of spikelets	–	25.3 ± 2.8	21.6 ± 2.9	20.4 ± 3.3	–	28.5 ± 1.9	23 ± 2.6	19.5 ± 0.7	–	–	22	-

*Data are shown for reproductive plants only, except for the final number of leaves in T0.

For the Bronsyn and Carvalis cultivars, the average heading date was postponed in relation to the duration spent in HT–SD conditions. Consequently, heading occurred later in T3 than in T2 and later in T2 than in T1. This delay was significant between all treatments in Bronsyn and only between T1–T3 and T1–T2 in Carvalis. Despite plants spending 6 weeks in HT–SD (T3), no heading was observed under short days, confirming that exposure to long photoperiods is necessary to trigger the floral transition. Interestingly, the delay between the transfer to HT–LD conditions and the heading date was not proportional to the duration of the HT–SD period (Fig. 2B). In fact, when calculating the heading date from the start of exposure to long photoperiods, the average delay to heading in Bronsyn was 56 days for T1, 42 days for T2, and 41 days for T3. A similar nonlinear response to the duration of exposure to long days was observed for Carvalis (T1: 77 days, T2: 54 days, and T3: 55 days).

### Final number of leaves and spikelets

**Figure 3.**
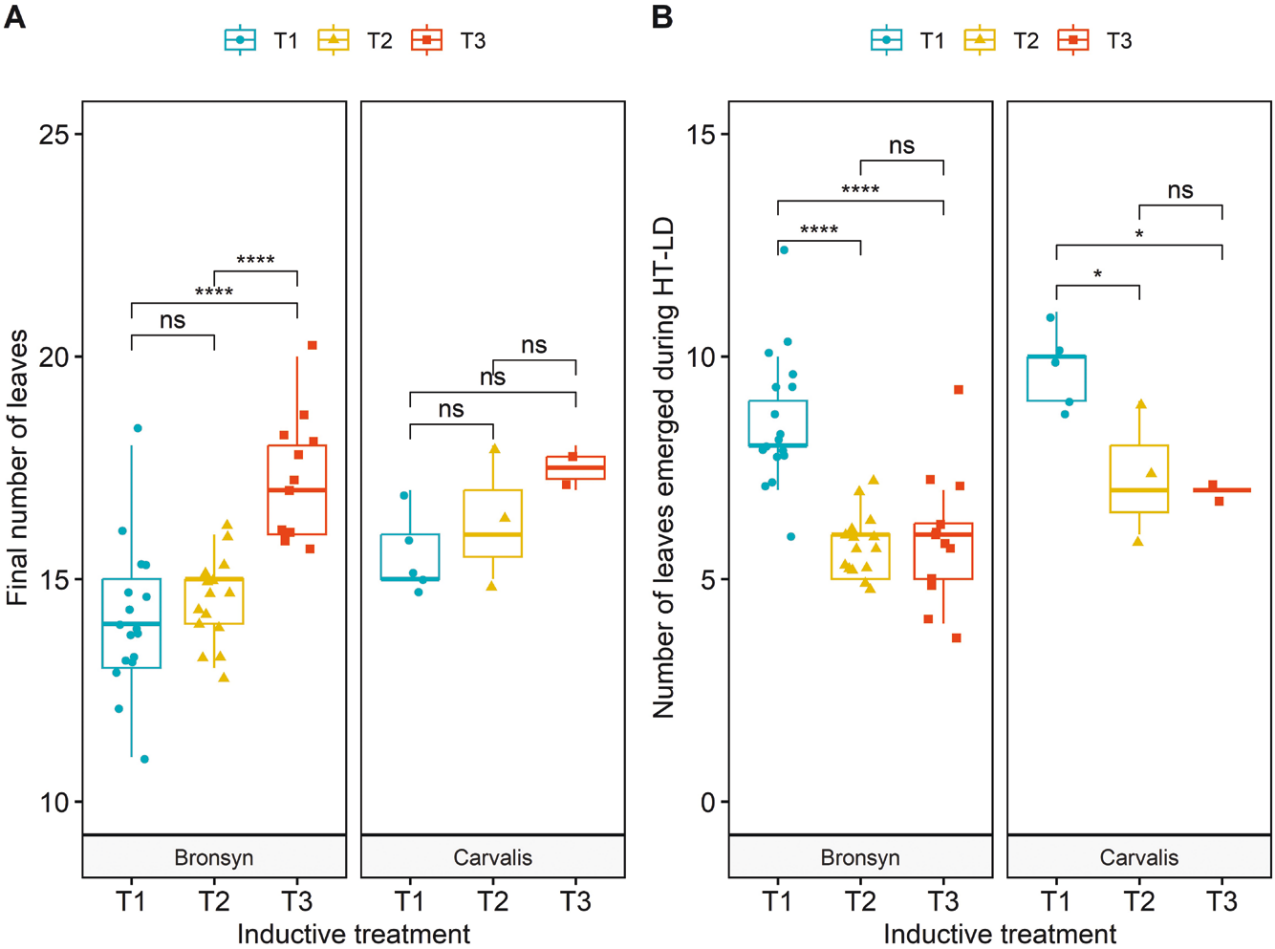
Final number of leaves (A) and number of leaves that have emerged since the transfer to high temperature-long day (HT–LD) conditions (B).Results are shown only for Bronsyn and Carvalis plants that reached the heading stage. The inductive treatments are denoted as T1 (blue circles), T2 (yellow triangles), and T3 (red squares). Statistical differences between treatments are shown in A (ns: non-significant, *P < 0.05, **P < 0.01, ***P < 0.001, ****P < 0.0001).

The final count of leaves on the headed tillers varied from 11 to 20 for Bronsyn and 15 to 18 for Carvalis. On average, the final leaf count for the headed tillers increased by three leaves between T1 and T3 in both cultivars (Fig. 3A), although statistical differences were only significant for T3 in Bronsyn. The number of leaves that emerged during the HT–LD period exhibited high variability across treatments and cultivars, ranging from 4 to 14 leaves (Fig. 3B). In T1, the number of leaves that emerged during the HT–LD period constituted 57% and 66% of the final leaf count for Bronsyn and Carvalis, respectively. For both cultivars, the number of leaves that emerged during this period was significantly higher in T1 compared to T2 and T3 (*T1–T2: W = 285.5, P < 0.001 and T1–T3: W = 198, P < 0.001 for Bronsyn; T1–T2: W = 14, P = 0.07, and T1–T3: W = 10, p < 0.05 for Carvalis*). There was no significant interactive effect between the varieties and the treatments (*P = 0.44*). For Bronsyn, T1 plants produced an average of two more leaves than T2 and T3 plants during the HT–LD period. The number of leaves that emerged during this period was identical between T2 and T3 treatments for Bronsyn (~6 leaves; *W* = 105.5, *P* > 0.05). For Carvalis, T1 plants produced an average of three more leaves than those of T2 and T3 during the HT–LD period.

**Figure 4.**
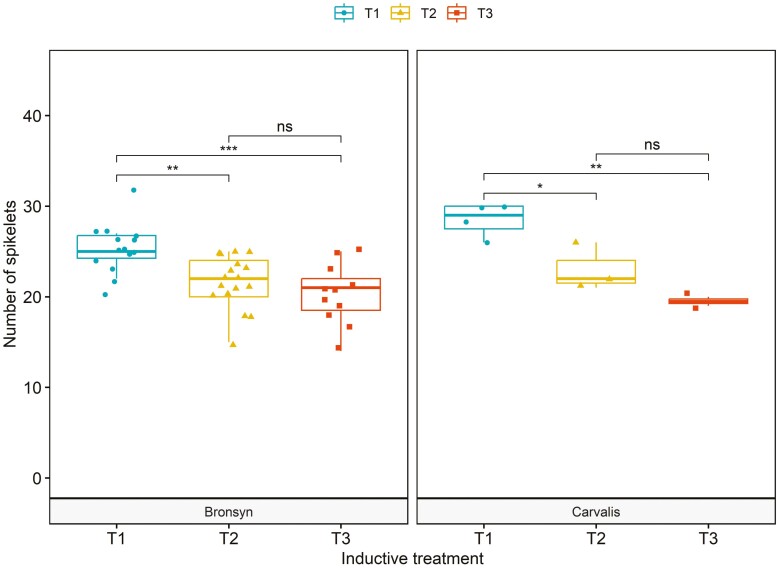
Number of spikelets per spike for cultivars Bronsyn and Carvalis. The inductive treatments are denoted as T1 (blue circles), T2 (yellow triangles), and T3 (red squares). Statistical differences between treatments are shown in A (ns: non-significant, *P < 0.05, **P < 0.01, ***P < 0.001, ****P < 0.0001).

The average number of spikelets per spike varied from 14 to 32 across treatments and cultivars (Fig. 4). The analysis of variance of spikelet number revealed neither significant differences between cultivars nor interactive effect between the varieties and the treatments (*P* = 0.33). Similar to the observation for leaves, the longer the exposure to HT–SD conditions, the fewer the spikelets. In Bronsyn, the number of spikelets was higher in T1 compared to T2 and T3 (*P* < 0.01). The differences between treatments were similar for Carvalis, with a decrease from 28 ± 1.7 spikelets in T1 to 19 spikelets in T3 (*P* < 0.01). Both the number of leaves that emerged during the HT–LD period and the number of spikelets per spike were significantly higher in T1 compared to T2 and T3.

### Leaf elongation rate and leaf ligulation rate

**Figure 5.**
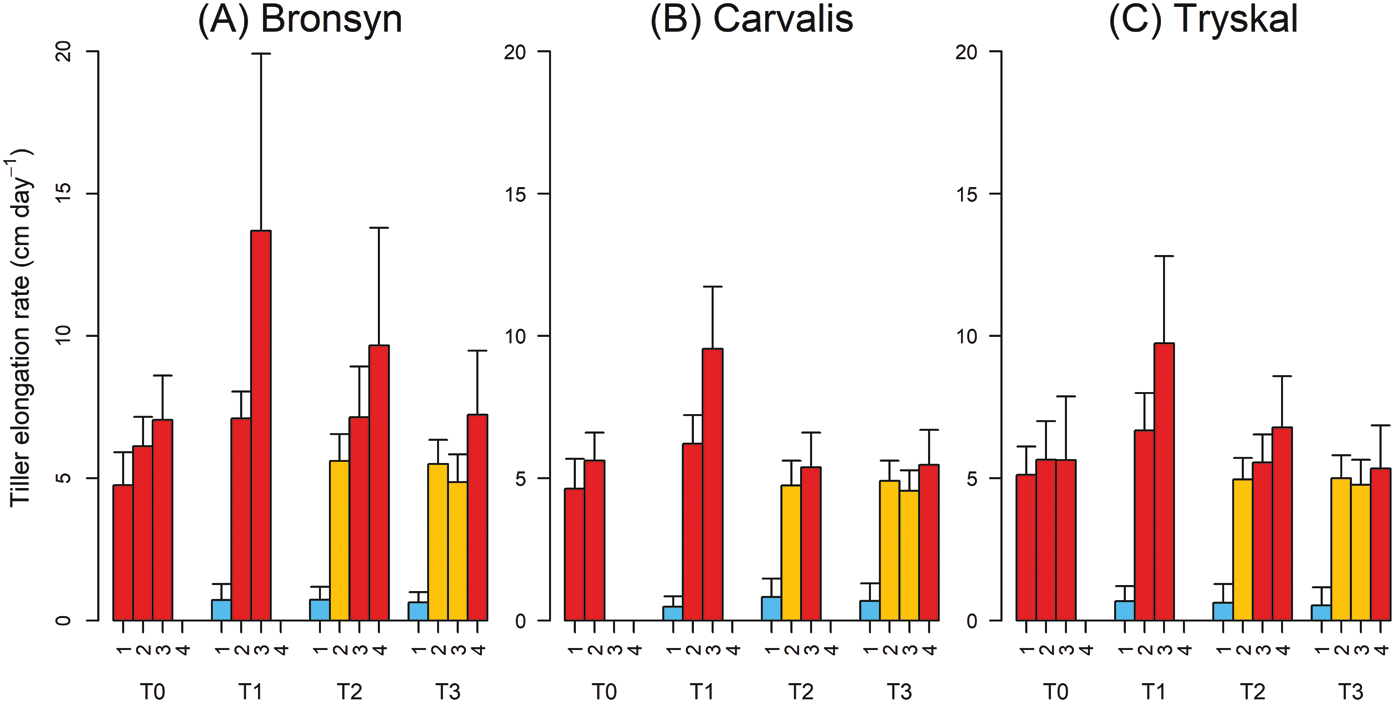
Dynamics of tiller elongation rate of cultivar Bronsyn (A), Carvalis (B), and Tryskal (C). Results are presented for each inductive treatment (T0, T1, T2, and T3). Based on the dynamics of individual leaf length, linear regressions were performed on the total length of the tillers, considering four 3-week periods corresponding to the treatment application schedule. For clarity, the low temperature–short day (LT–SD) conditions are represented by blue bars, high temperature–short day (HT–SD) by orange bars, and high temperature–long day (HT–LD) by red bars. The value of the bars is the mean + SD.

Based on individual leaf length dynamics, linear regressions were performed on the total tiller length (sum of all leaf lengths) across four 3-week periods corresponding to the treatment application schedule (Fig. 5). For T0 plants, exposed solely to HT–LD conditions, tiller elongation rates tended to increase during the experiment, particularly for Bronsyn (*period 1–2: W = 99, P < 0.01 and period 1–3: W = 71, P < 0.01*). After three weeks in T0 conditions, Bronsyn’s elongation rate was significantly higher than that of Carvalis (*P < 0.01*) and similar to Tryskal (*P = 0.08*). For T1, T2, and T3 plants, a notably low leaf elongation rate was observed during exposure to low temperature (LT–SD) (mean *±* SD of all cultivars = 0.54 cm day^−1^ ± 0.04) with no significant difference between treatments (*P* = 0.42) or varieties (*P* = 0.65). The leaf elongation rate then sharply increased to 5.4 cm day^−1^ ± 0.08 when the plants were transferred to high temperature (HT–SD and HT–LD). The leaf elongation rate of tillers exposed to HT remained remarkably stable as long as plants were not transferred to long day conditions (HT–SD; orange bars). In these HT-SD conditions, plants of cultivar Bronsyn showed the highest rate of leaf elongation compared to Carvalis and Tryskal (*P* < 0.001). For T1 and T2 plants, leaf elongation rate increased for several weeks after transfer to HT–LD. For all cultivars, exposure to long days and high temperature immediately after the LT–SD period (T1 period 2; red bars in Fig. 5) significantly (*P* < 0.001) increased the rate of leaf elongation compared to plants exposed to high temperature but remaining under short days (T2 period 2 and T3 periods 2–3; orange bars in Fig. 5). The same dynamics were observed when T2 and T3 plants were transferred to long days (T2, period 2 vs. 3: *P* < 0.01, except for Tryskal; T3, period 3 vs. 4: *P *< 0.05). For T1, T2, and T3, the maximal leaf elongation rate was delayed approximately proportionally to the time spent under HT–SD (during period 3 for T1, during period 4 for T2 and later for T3). In Carvalis and Tryskal cultivars, which had few reproductive tillers, the mean acceleration of leaf elongation was significantly lower than for Bronsyn, especially in T2 and T3. The above analyses of variance revealed no significant effect between the varieties and the treatments.

**Figure 6.**
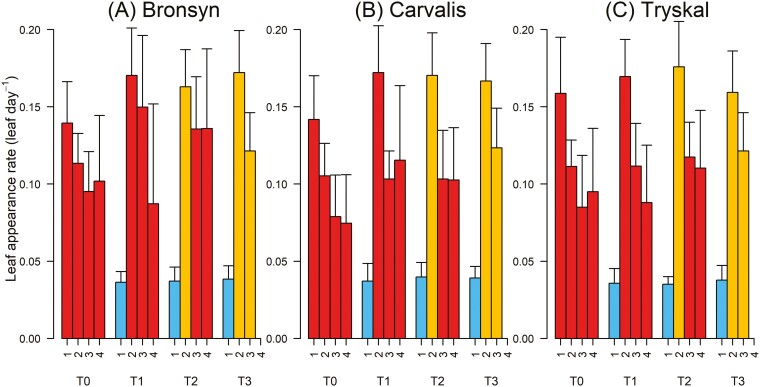
Dynamics of leaf appearance of cultivars Bronsyn (A), Carvalis (B), and Tryskal (C). Results are presented for each inductive treatment (T0, T1, T2, and T3). Linear regressions were performed on the total length of the tillers, considering four 3-week periods corresponding to the treatment application schedule. For clarity, the low temperature–short day (LT–SD) conditions are represented by blue bars, high temperature–short day (HT–SD) by orange bars, and high temperature–long day (HT–LD) by red bars. The value of the bars is the mean + SD.s

The impact of the inductive treatments on the dynamics of the leaf appearance rate (LAR) exhibited similarity across the three cultivars (Fig. 6). For T0 plants, consistently exposed to HT–LD throughout the experiment, the LAR gradually declined from approximately 0.146 to 0.09 leaves per day (l day^−1^) over four weeks for all cultivars. During their exposure to low temperature (LT–SD), plants of T1, T2, and T3 demonstrated a notably low LAR of about 0.035 l day^−1^ which was similar across all cultivars and treatments (*P* = 0.22 and 0.35, respectively). This rate significantly increased to around 0.16 l day^−1^ when plants were exposed to high temperatures (*P* < 0.001), subsequently decreasing progressively as the tillers aged across all cultivars and treatments. Contrary to the results shown for leaf elongation rate (Fig. 5), the photoperiod did not influence LAR, as no significant differences were observed among T1, T2, and T3 (period 2: *P* = 0.54).

## Discussion

### A dual floral induction but different requirements among cultivars

In *L. perenne*, it has been demonstrated that floral induction requirements vary across populations and cultivars (Heide 1994; Rouet *et al.* 2021). Within this intraspecific diversity, certain populations, typically found in the Mediterranean region, appear to have a unique induction phase, as no specific cold temperature requirements were identified (Aamlid *et al.* 2000). In this study, we selected cultivars adapted to temperate climates, characterized by cool winters and moderately dry summers. Consequently, the cultivars under study were anticipated to exhibit a dual floral induction, comprising a primary and a secondary induction (Heide 1994). Our findings corroborate this hypothesis, as monitoring of heading dates and tiller dissections revealed that without exposure to low temperatures and short photoperiods (T0; LT–SD), all tillers remained vegetative and produced up to 20 leaves. Although the proportion of tillers reaching the reproductive stage varied significantly among cultivars, floral transition, and heading were only observed following successive exposure to LT–SD and HT–LD conditions. These observations confirm the requirement of low temperatures followed by long days for floral induction in the studied cultivars. However, to strictly require long photoperiods, a fifth treatment combining LT–SD followed by HT–SD throughout the entire experiment would have been necessary.

While this study did not aim to establish quantitative responses of primary and secondary inductions to temperature and photoperiod, the low proportion of heading plants in Carvalis and Tryskal cultivars prompted questions about the inductive requirements for floral transition in these plants. The photoperiod used for inducing secondary induction (16 hours) exceeded that found in France, where the studied cultivars regularly reach reproductive stages. For instance, in Lusignan (latitude: 46.4333 and longitude: 0.1167), the maximum photoperiod is 15.8 hours at the summer solstice, which is long after the observed heading dates. The average temperature set at 18°C in the HT–LD condition was also higher than that observed in France during the completion of secondary induction (~late winter-early spring). As for primary induction, the LT–SD condition was expected to fulfil its completion for all cultivars compared to the natural conditions observed in the GEVES trials in winter (Aamlid *et al.* 2000; Paina *et al.* 2014). The photoperiod (8 hours) was shorter than the minimum photoperiod in Lusignan (8.6 hours at the winter equinox and minimum day length) and the temperature was equivalent to the mean winter temperature in Lusignan (5°C compared to 5.1°C). Lastly, the duration of the low temperature period (9 weeks) surpassed the average number of days with a mean temperature below 5°C in Lusignan (8 weeks on average between 15th September and 30th July for the period 2001–2016). Interestingly, a recent experiment we conducted at Lusignan (unpublished data) revealed that Bronsyn plants grown outdoors had more reproductive tillers (15%) than Tryskal (~2%) after one year.

The low proportion of heading in Carvalis and Tryskal could be attributed to the existence of a minimum developmental stage, after which plants become competent for primary induction (Cooper 1960; Bommer 1961; Heide 1994). However, this seems unlikely as the tillers exposed to LT–SD conditions were beyond the 3-leaf stage, while primary induction in *L. perenne* has been shown to respond at earlier stages (Heide 1994). Another plausible explanation for the low proportion of heading could be the absence of extremely low temperatures (<5°C). Indeed, brief and infrequent episodes of very low temperatures might exert a stronger effect than a prolonged exposure to temperatures near 5°C. Cooper (1960) also observed a high variability in the proportion of heading plants from the *Lolium* genus (ranging from 0 to 81%). The author hypothesised that this variability was due to an incomplete primary induction resulting from the insufficient inductive effect of the cold conditions used (germinated seeds for 0, 4, and 8 weeks in a refrigerator at 0*–*3°C).

### A putative effect of plant size on heading dynamics

As anticipated, our findings indicated that the earlier the plants were exposed to long days post-primary induction, the sooner the heading date. However, when expressing the heading date as the number of days since exposure to long days, we discovered that plants kept under HT–SD conditions reached the heading stage earlier than those transferred to long days immediately postprimary induction. This relationship between the duration of HT–SD conditions and the heading time postexposure to long days was nonlinear. Moreover, our results demonstrated that the reduction in time to heading (expressed from exposure to long days) occurred in plants with a higher leaf count, attributable to these plants remaining vegetative for an extended period (HT–SD). The present results also showed that the average leaf count of tillers on the day of the transfer to LD was higher in T2 (~ +50%) and T3 (~ +83%) than in T1 (*P* < 0.001; Fig. 3, Table 2)). We also observed that when the plants of T2 and T3 were exposed to long days, the delay to heading was reduced compared to T1 as well as the subsequent initiation of leaves and spikelets. These findings strongly suggest that the rate of secondary induction is positively influenced by tiller size and/or the number of leaves. The apices of larger plants may be more responsive to the inductive conditions (long days in this case), thereby swiftly transitioning from leaf production to the production of reproductive organs. Cooper (1960) observed similar relationships between heading dates and the number of leaves emerging under continuous days in several temperate cultivars of *L. perenne*.

### Acceleration of leaf and spikelet production under long day conditions

During floral induction, the terminal apex of tillers undergoes significant transformations, marking the shift from the vegetative to the reproductive stage (Gillet 1980; Thomas 1980). The precise timing and processes driving the conversion of primordia into leaves or spikelets remain subjects of ongoing research. A consensus among many researchers is that the general structure of the apex remains nearly invariant in terms of size, mitotic index, and number of primordia during the vegetative stages (Gillet 1980; Gonthier and Francis 1989; Kemp *et al.* 1989). Gonthier and Francis (1989), who conducted tiller dissections following exposure to various inductive treatments, discovered that this seemingly permanent state persisted during the primary induction. This study found similar results when observing the apex of plants cultivated under non-inductive conditions (data not shown). The consistent apex structure and morphology during vegetative stages likely result from a coordination between the initiation of primordia and their differentiation into leaves.

The leaf appearance rate (LAR) did not appear to respond to day length, even seeming to decrease over time under LD conditions, contradicting data on primordia production (Canode *et al.* 1972; Kemp *et al.* 1989). However, it is possible to discern consistent impacts of day duration conditions by examining the balance of initial and final numbers of leaves and spikelets observed during the LD exposure period. Under the conservative hypothesis that (i) two growing leaves remained inside the sheath of older leaves and (ii) the primordia production rate during the vegetative stage is approximately the same as the LAR, the following numbers were inferred. Approximately three leaves, i.e. three primordia, were likely produced during the SD periods for T2 and T3, with a rate of 0.14 primordium per day. Based on the total number of leaves that appeared during the LD period plus the number of spikelets, and assuming the spikelet and primordia production rates are similar, we can estimate that the primordia production rate under LD conditions was approximately 0.53 and 0.64 primordia per day in T1 and T2 or T3, respectively. This rate is, on average, ~four-fold the rate estimated under SD conditions. Such acceleration aligns with figures published earlier in grasses (Canode *et al.* 1972; Gonthier and Francis 1989; Kemp *et al.* 1989), including cereals (Jamieson *et al.* 1995).

### Leaf elongation rate and floral induction

For plants in T2 and T3, the initial acceleration of leaf elongation was attributed solely to the effect of temperature, while the more pronounced increase in leaf elongation rate for T1 plants resulted from the combined effects of temperature and photoperiod increases. This acceleration of leaf elongation around the time of floral transition aligns with our observations and has been consistently reported in literature (Davies 1978; Parsons and Robson 1980). Parsons and Robson (1980) proposed a causal link between the increase in leaf elongation rate and the floral transition. Conversely, Davies (1971) suggested that the acceleration of leaf elongation was independent of the floral transition and was driven by the same environmental factors as the floral induction, i.e. the photoperiod. To properly verify the independence of the effects of photoperiod and floral transition on leaf elongation rate, an additional treatment involving plants exposed to high temperature and short photoperiod before being transferred to high temperature and long photoperiod would have been necessary. However, our results indicated that the leaf elongation rate of plants in short days (HT–SD in T2 and T3) was slower than that of plants immediately transferred to long days (HT–LD in T1). This suggests that the floral transition may have an additive effect on the increase in leaf elongation induced by exposure to long days. The underlying mechanisms by which the floral transition or the photoperiod itself controls leaf elongation remain unclear to date but they may involve major phytohormones. For instance, it has been shown that KNOX proteins are key players in auxin, gibberellin, and cytokinin biosynthesis in the meristems during floral transition and inflorescence morphogenesis (McSteen and Leyser 2005; Colasanti and Coneva 2009; Zhang and Yuan 2014). At the same time, it appears that the same set of hormones actively regulates the rate of cell division in the leaf growth zones of maize plants (De Vos *et al.* 2020).

## Conclusion

Perennial ryegrass demonstrated significant interactions between leaf growth and reproductive development, influenced by both genetic and environmental factors. Our findings confirmed that the floral transition only occurs following sequential exposure to low temperatures and long photoperiods even for the cultivars with low proportions of reproductive tillers. We noted that the date of exposure to a long photoperiod impacted the final count of leaves and spikelets. Additionally, the rate of leaf and spikelet production noticeably escalated following the plants’ exposure to long photoperiods. Our results also suggest a direct, positive influence of photoperiod on leaf elongation rate, an effect that is further amplified in plants undergoing the floral transition. Finally, this work demonstrated original GxE interactions between the vegetative and reproductive development with strong consequences on plant fitness.

## Supplementary Material

plae069_suppl_Supplementary_Material

## Data Availability

The data underlying this article are available in Recherche Data Gouv, at https://doi.org/10.57745/MJEKAZ
