# Supplementary material for: Experimental analysis of genetic and environmental interactions on leaf elongation and reproductive development in Lolium perenne
Source: AoB Plants. 2024 Dec 24;17(1):plae069. doi: 10.1093/aobpla/plae069 (PMC11756295; doi:10.1093/aobpla/plae069)
Supplement: plae069_suppl_Supplementary_Material [file plae069_suppl_supplementary_material.pdf]

SUPPLEMENTARY MATERIAL

A

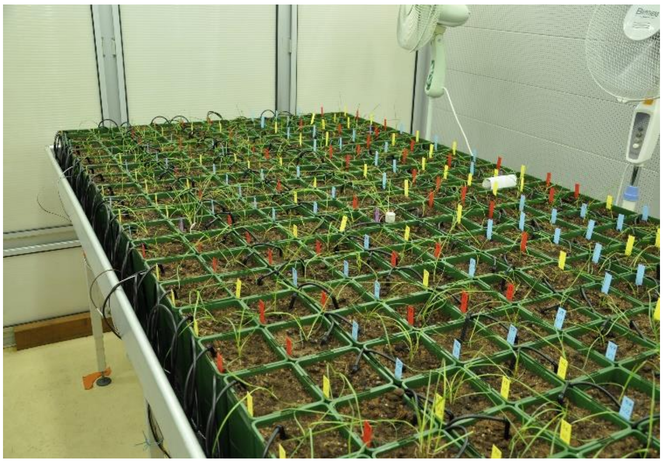

B

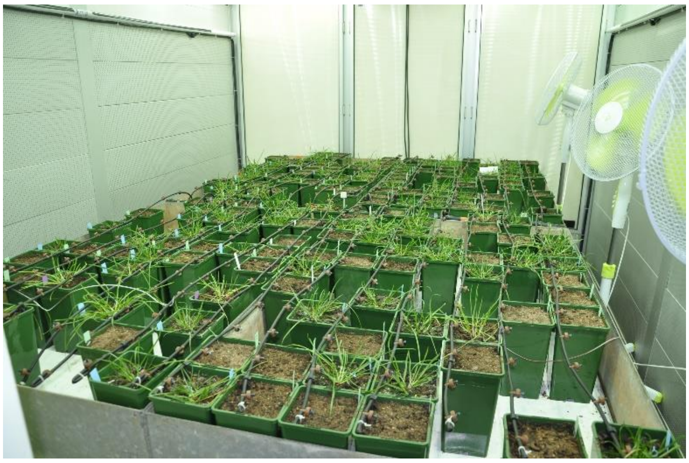

C

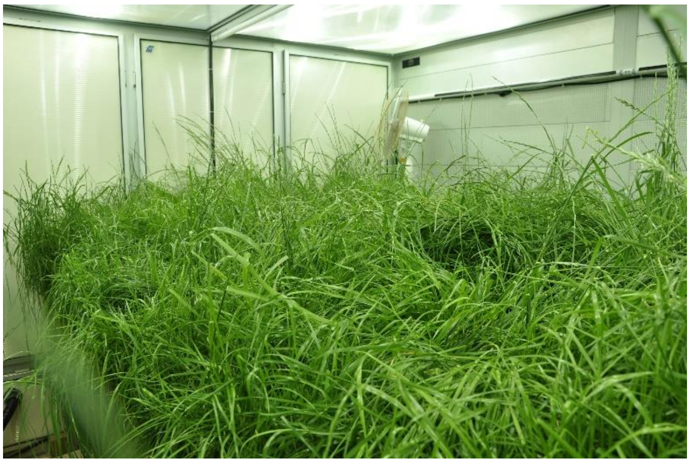

Figure S1. Experimental setup. A: plants of treatments 1, 2 and 3 during primary induction (low temperature and short days). B: plants of treatment 0 maintained in

non-inductive conditions (high temperature and short days). C: Plants of treatments 0, 1, 2 and 3 at heading.

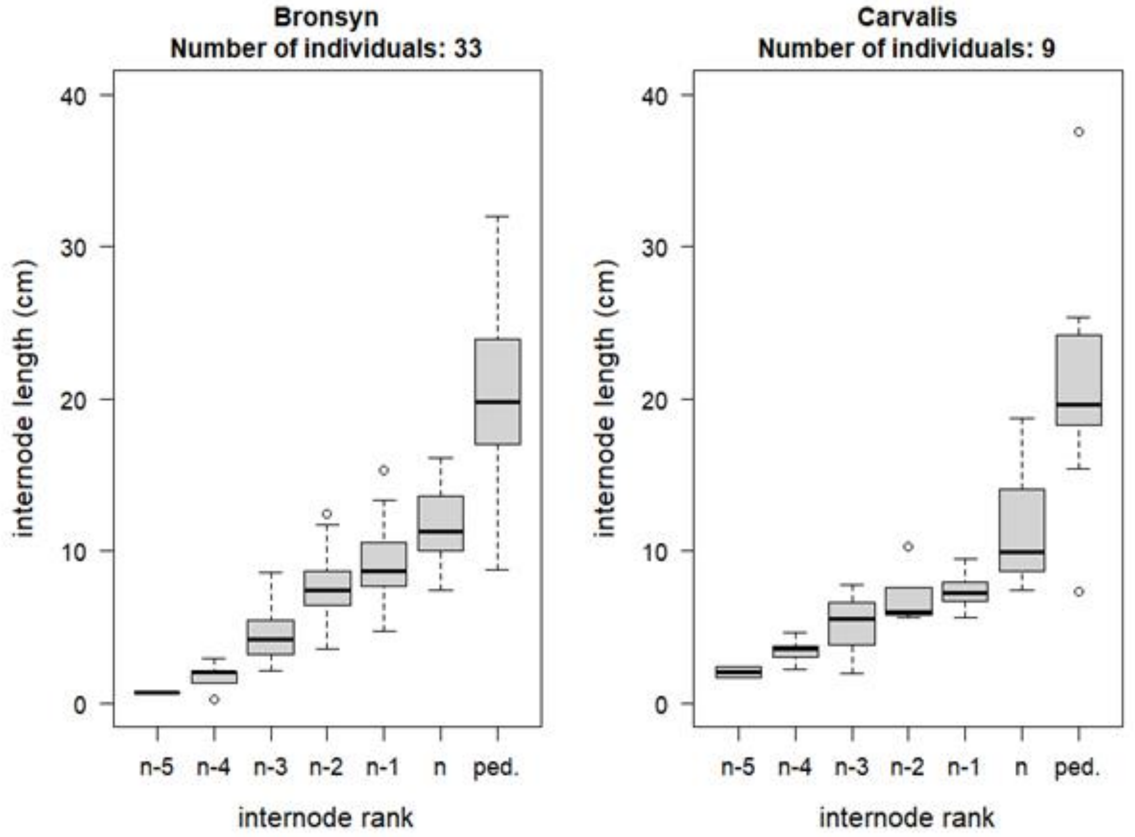

Figure S2. Length of internodes depending on their rank for cultivars Bronsyn, Carvalis exposed to treatment T1. Three tillers per plant (including the main tiller) were measured. Internode rank is given relatively to the last internode. “ped.” corresponds to the peduncle.

For Bronsyn and Carvalis, tillers had at 3 to 6 long internodes in addition to the peduncle (Figure S2). In average, Bronsyn exhibited 5.2 ±0.7 long internodes against 5.8 ±0.8 in Carvalis. Internodes *n*-5, *i.e.* the oldest ones, were slightly elongated (0.7 cm and 2.0 ±0.5cm in Bronsyn and Carvalis, respectively) and only observed in 3% of Bronsyn’s tillers against 22% in Carvalis. In contrast, internodes *n-3* were elongated in 88 and 100% of the tillers of Bronsyn and Carvalis, respectively. The slightly higher number of internodes observed in Carvalis was associated with smaller internodes compared to Bronsyn, thus leading to similar total length of tillers at maturity (52.9 ±9.3 cm for Bronsyn and 54.0 ±11.6 cm for Carvalis). In every plants of both cultivars, peduncles were significantly longer than the other internodes and represented 27 ±5% of the total length of the culm.

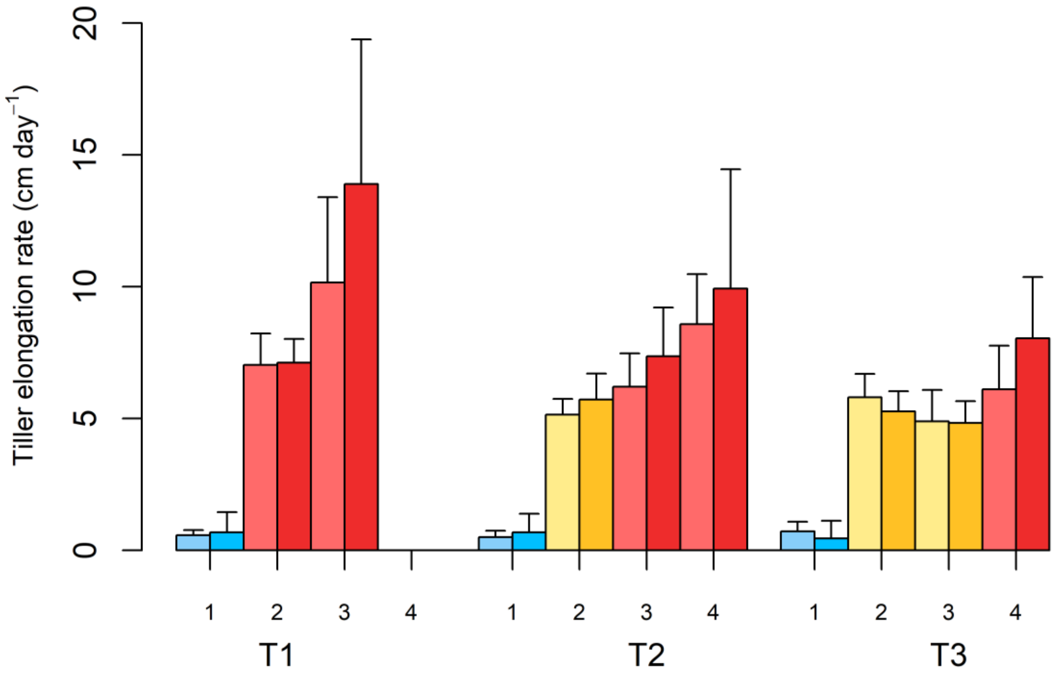

Figure S3: Dynamics of tiller elongation rate of cultivar Bronsyn for the inductive treatments T1, T2 and T3. Results are presented for plants that remained vegetative during the experiment (light colours) and those that turned reproductive (dark colours). The LT–SD conditions are represented by blue bars, HT–SD by orange bars and HT-LD by red bars. Value of the bars is the mean + SD.
